# Supplementary material for: Could Chronic Rhinosinusitis Increase the Risk of Ulcerative Colitis? A Nationwide Cohort Study
Source: Diagnostics (Basel). 2022 Sep 28;12(10):2344. doi: 10.3390/diagnostics12102344 (PMC9600918; doi:10.3390/diagnostics12102344)
Supplement: Supplementary file 1 [file diagnostics-12-02344-s001.zip › diagnostics-1934709-supplementary.pdf]

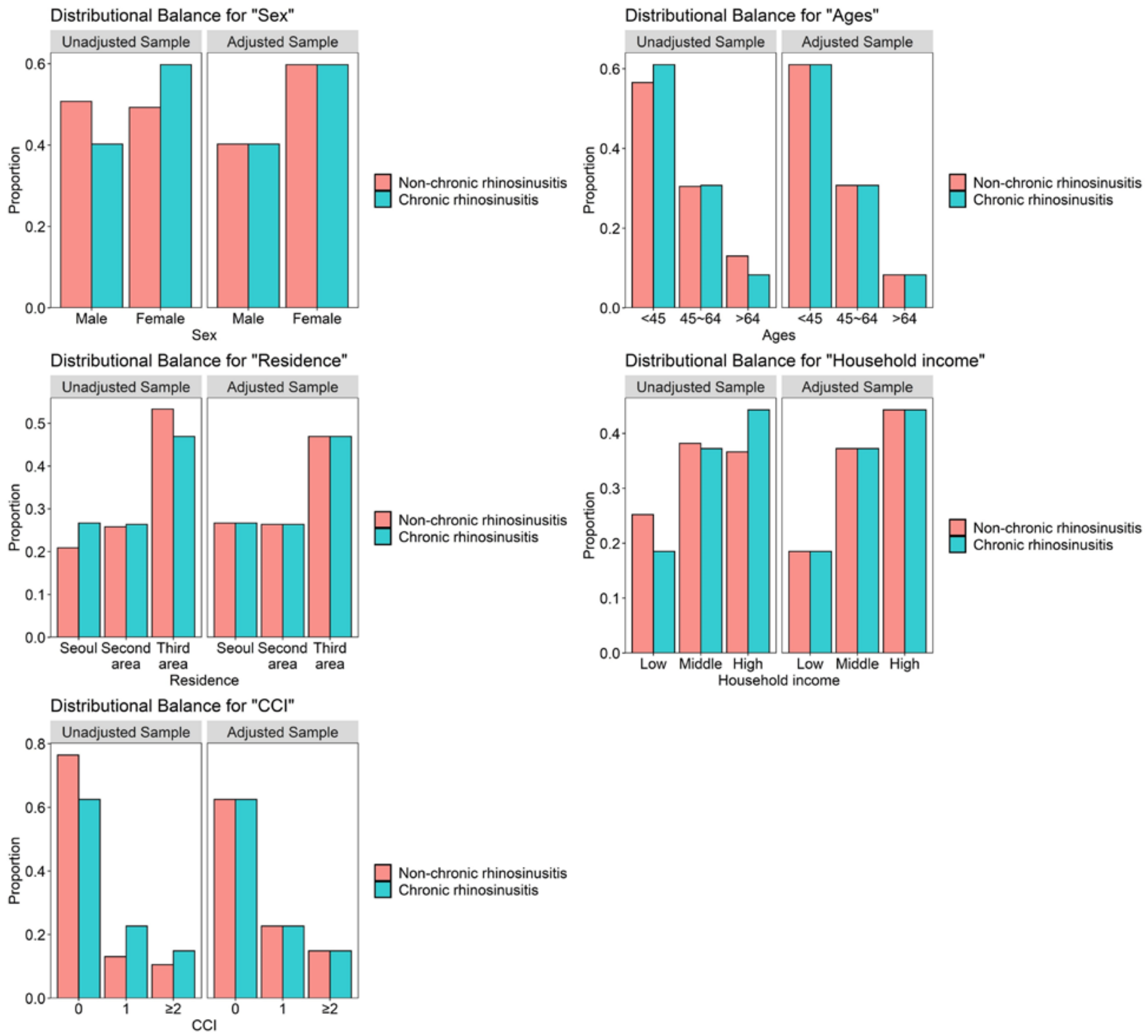

Figure S1. Confirmation of appropriate matching between the comparison and chronic rhinosinusitis groups using the balance plot for five variables before and after matching.
